# Mechanistic study of mtROS-JNK-SOD2 signaling in bupivacaine-induced neuron oxidative stress

**DOI:** 10.18632/aging.103447

**Published:** 2020-04-27

**Authors:** Zhongjie Liu, Shiyuan Xu, Zhonghua Ji, Huali Xu, Wei Zhao, Zhengyuan Xia, Rui Xu

**Affiliations:** 1 Department of Anesthesiology, Zhujiang Hospital, Southern Medical University, Guangzhou, Guangdong Province, China; 2 Department of Anesthesiology, Affiliated Zhuhai Hospital of Jinan University, Zhuhai, Guangdong Province, China; 3 Department of Anesthesiology, University of Hong Kong, Pokfulam, Hong Kong, China

**Keywords:** reactive oxygen species, manganese superoxide dismutase, bupivacaine, oxidative stress, apoptotic injury

## Abstract

Manganese superoxide dismutase (SOD2) is a key enzyme to scavenge free radical superoxide in the mitochondrion. SOD2 deficiency leads to oxidative injury in cells. Bupivacaine, a local anesthetic commonly used in clinic, could induce neurotoxic injury via oxidative stress. The role and the mechanism of SOD2 regulation in bupivacaine-induced oxidative stress remains unclear. Here, bupivacaine was used to treat Sprague-Dawley rats with intrathecal injection and culture human neuroblastoma cells for developing vivo injury model and vitro injury model. The results showed that bupivacaine caused the over-production of mitochondrial reactive oxygen species (mtROS), the activation of C-Jun N-terminal kinase (JNK), and the elevation of SOD2 transcription. Decrease of mtROS with N-acetyl-L-cysteine attenuated the activation of JNK and the increase of SOD2 transcription. Inhibition of JNK signaling with a small interfering RNA (siRNA) or with sp600125 down-regulated the increase of SOD2 transcription. SOD2 gene knock-down exacerbated bupivacaine-induced mtROS generation and neurotoxic injury but had no effect on JNK phosphorylation. Mito-TEMPO (a mitochondria-targeted antioxidant) could protect neuron against bupivacaine-induced toxic injury. Collectively, our results confirm that mtROS stimulates the transcription of SOD2 via activating JNK signaling in bupivacaine-induced oxidative stress. Enhancing antioxidant ability of SOD2 might be crucial in combating bupivacaine-induced neurotoxic injury.

## INTRODUCTION

Reactive oxygen species (ROS), as a byproduct of oxidative phosphorylation, are mainly produced in the mitochondria [1, 2]. Enough evidence has revealed that ability deficiency of scavenging ROS leads to the damage of mitochondrial lipid membrane, the release of mitochondrial cytochrome c and the activation of mitochondrial death pathway [3, 4]. With reports of cauda equine syndrome and transient neurological symptoms following continuous spinal anesthesia or high concentration of local anesthetic application, more and more clinicians pay attention to the local anesthetic-induced neurotoxic injury [5, 6]. Bupivacaine (BPV), an amide compound, is widely administrated for regional nerve block and analgesia [7]. It uncouples oxidative phosphorylation, inhibits ATP production, and collapses the mitochondrial membrane potential [8]. The decrease of ATP activates adenosine 5´-monophosphate (AMP)-activated protein kinase signaling which results in a marked increase of intracellular ROS. We have demonstrated that oxidative stress-mediated apoptosis is a crucial mechanism of BPV-induced neuron injury [9].

Manganese superoxide dismutase (SOD2) is the essential mitochondrial antioxidant enzyme that detoxifies the free radical superoxide in mammalian cells [10, 11]. It transfers highly reactive O_2_^−^ into H_2_O_2_, which is reduced to H_2_O in the mitochondria [12]. The homeostasis balance in the activation of SOD2 and the production of free radical superoxide determine whether cells suffer from oxidative stress and apoptosis. SOD2 deficiency leads to mitochondrial oxidative stress and glycation of mitochondrial DNA, which plays a crucial role in mitochondria oxidative stress and neuron apoptosis [13, 14]. The interaction between SOD2 transcription and ROS production is different in some pathological processes. SOD2 deficiency reportedly exacerbates the mitochondrial ROS (mtROS) over-production and oxidative damage in Chagas disease [15]. At the same time, previous evidence demonstrates that ROS stimulates SOD2 expression through activation of p53 [16]. The mechanism is that ROS drives the nucleus translocation of extracellular regulated protein kinases, where it phosphorylated p53 at Ser15, leading to the activation of p53 and subsequent up-regulation of SOD2 transcription [17]. However, the role and transcription of SOD2 in BPV-induced oxidative stress remains unclear.

C-Jun N-terminal kinase (JNK) is a stress-inducible kinase in response to various extracellular and intracellular nociceptive stimulus, such as oxidative damage, UV light, chemicals and biological agents [18–20]. When phosphorylated, JNK signaling is activated to change stress related proteins transcription for modulating cell survival or death [21, 22]. Whether or not SOD2 transcription is regulated through mtROS-driven activation of JNK signaling in oxidative stress has not been reported.

In this study, BPV was used to treat Sprague-Dawley rats with intrathecal injection and culture human neuroblastoma (SH-SY5Y) cells for developing vivo injury model and vitro injury model. This study may elucidate the mechanism of mtROS-JNK-SOD2 signaling in BPV-induced neuron oxidative stress and provide promising therapy for above neurotoxic injury.

## RESULTS

### BPV induced spinal reflex dysfunction and apoptotic injury in vivo

**Figure 1:**
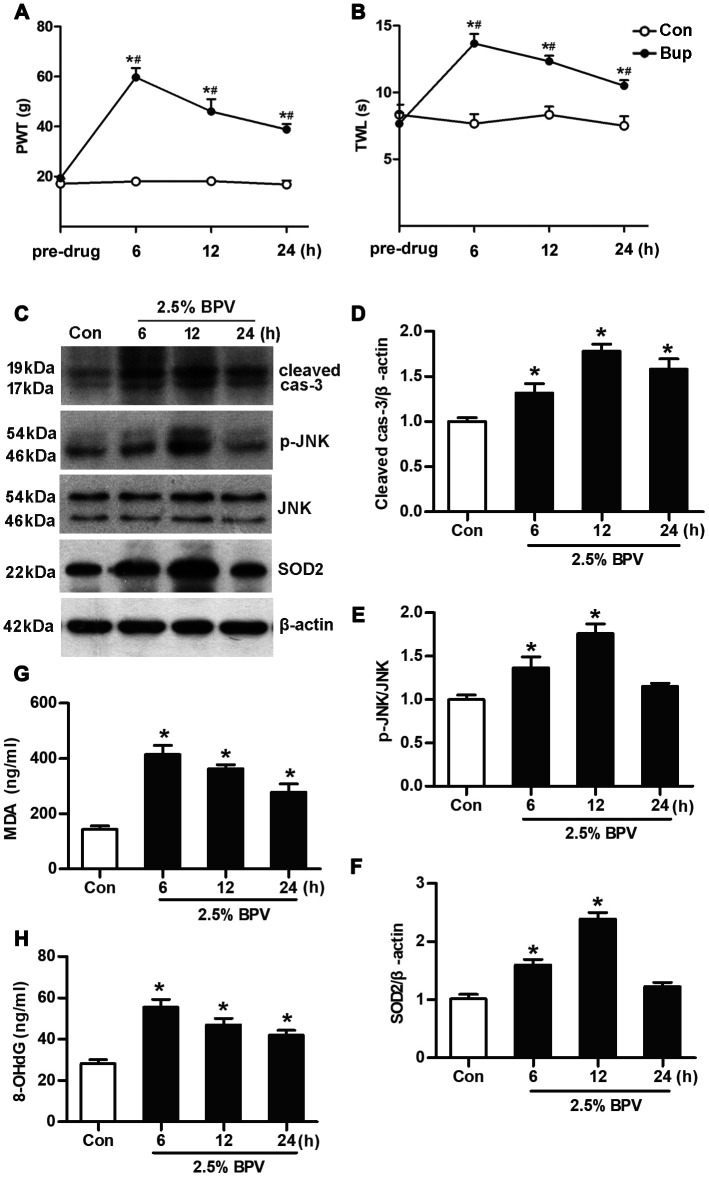
**BPV caused spinal cord oxidative injury to activate JNK signaling and elevate SOD2 transcription in rats.** Con: intrathecal injection of 0.9% saline with 0.2 μl/g in rats; BPV: intrathecal injection of 2.5% BPV with 0.2 μl/g in rats. (**A**, **B**) spinal reflex function in different times (6^th^ h, 12^th^ h or 24^th^ h after intrathecal injection of 2.5% BPV or 0.9% saline with 0.2 μl/g) was investigated by PWT and TWL values; pre-drug: rats before intrathecal injection of 0.9% saline or 2.5% BPV; Values are the mean± SEM of n = 6; *: *P*< 0.05 compared with the pre-drug; #: *P*< 0.05 compared with group Con. (**C**–**H**) MDA and 8-OHdG production, JNK phosphorylation and SOD2 transcription were measured in spinal intumescentia lumbalis of rats in different times (6^th^ h, 12^th^ h or 24^th^ h after intrathecal injection of BPV or saline); Values are the mean± SEM of n = 6; *: *P*< 0.05 compared with the group Con.

Spinal reflex function was assessed by paw withdrawal threshold (PWT, g) and thermal withdrawal latency (TWL, s). They were tested in different times (pre-drug, 6^th^ h, 12^th^ h and 24^th^ h) after rats with intrathecal injection of 2.5% BPV. In group BPV, PWT and TWL values were significantly elevated in 6^th^ h, 12^th^ h and 24^th^ h after injection (vs. pre-drug, *P*< 0.05). PWT and TWL values were also significantly elevated in 6^th^ h, 12^th^ h and 24^th^ h after intrathecal injection of BPV (group BPV vs. group Con, *P*< 0.05). (Figure 1A, 1B).

Spinal cord apoptotic injury was determined with cleaved caspase-3 expression. They were measured in different times (pre-drug, 6^th^ h, 12^th^ h and 24^th^ h) after rats with intrathecal injection of 2.5% BPV. Cleaved caspase-3 expression was significantly elevated in 6^th^ h, 12^th^ h and 24^th^ h after intrathecal injection of BPV (vs. group Con, *P*< 0.05, Figure 1C, 1D).

### BPV caused oxidative injury, activated JNK signaling and elevated SOD2 transcription in vivo

After intrathecal injection of 2.5% BPV, JNK phosphorylation and SOD2 expression were elevated in 6^th^ h and 12^th^ h in spinal cord of rats (vs. pre-drug, *P*< 0.05, Figure 1C–1F). Spinal cord oxidative injury was measured with malondialdehyde (MDA) and 8-hydroxydeoxyguanosine (8-OHdG) generation. MDA and 8-OHdG production significantly were increased in 6^th^ h, 12^th^ h and 24^th^ h after intrathecal injection of 2.5% BPV in spinal cord of rats (vs. group Con, *P*< 0.05, Figure 1G, 1H).

### mtROS-JNK-SOD2 signaling was activated in BPV-induced vitro injury model

**Figure 2:**
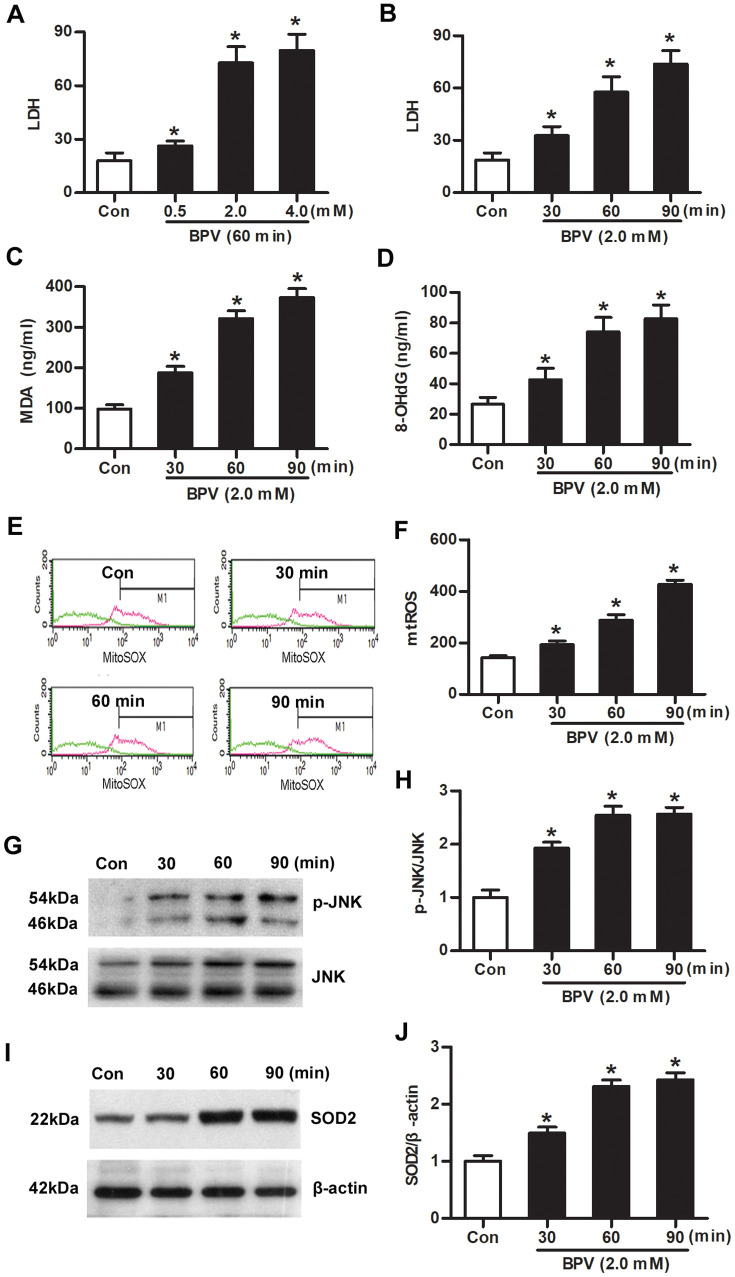
**Oxidative injury, JNK phosphorylation and SOD2 transcription were elevated in SH-SY5Y cells treated with BPV.** (**A**) LDH in cells treated with different concentration (0.5, 2.0, 4.0 mM) of BPV for 60 min; (**B**) LDH in cells treated with 2.0 mM BPV for 30, 60, or 90 min.; (**C**, **D**) MDA and 8-OHdG production in cells treated with 2.0 mM BPV for 30, 60, or 90 min; (**E**, **F**) the fluorescence intensities of mtROS in cells incubation with 2.0 mM BPV for 30, 60, or 90 min; (**G**, **H**) the western blot analysis shows JNK and p-JNK in SH-SY5Y cells incubation with 2.0 mM BPV for 30, 60, or 90 min; (**I**, **J**) the western blot analysis showed SOD2 expression in cells incubation with 2.0 mM BPV for 30, 60, or 90 min. Values are the mean± SEM of n = 3. *: *P*< 0.05 compared with the group Con.

Cells were cultured with different BPV concentrations (0.5, 2.0, or 4.0 mM) for 60 min. Cytotoxicity was measured with lactate dehydrogenase (LDH) assays. LDH production was increased in cells cultured with 0.5, 2.0, or 4.0 mM BPV (vs. group Con, *P*< 0.05, Figure 2A).

Next, cells were cultured with 2.0 mM BPV for 30, 60, or 90 min. Oxidative injury (LDH, MDA, 8-OHdG and mtROS generation), JNK phosphorylation and SOD2 expression were measured. Oxidative injury was significantly elevated in cells treated with BPV in 30, 60, or 90 min (vs. group Con, *P*< 0.05, Figure 2B–2D). BPV stimulated an increase of JNK phosphorylation and SOD2 transcription that paralleled mtROS generation (vs. group Con, *P*< 0.05, Figure 2E–2J).

### SOD2 transcription was up-regulated via mtROS-JNK signaling in BPV-induced vitro oxidative injury model

**Figure 3:**
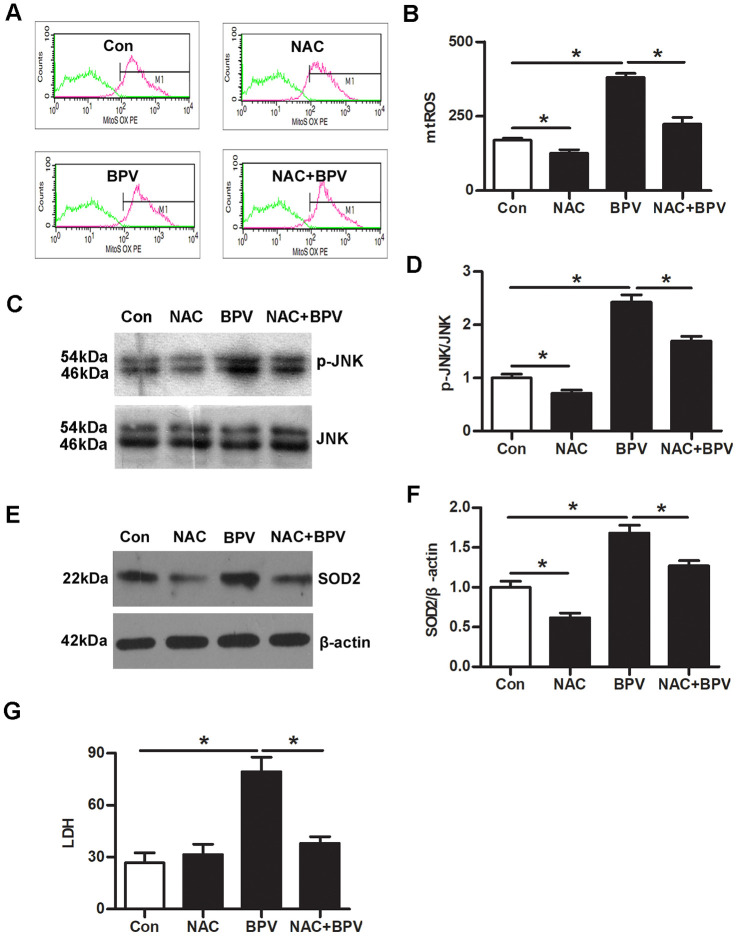
**mtROS activated JNK signaling and stimulated SOD2 transcription in BPV-induced neuron oxidative stress.** Con: untreated cells; NAC: cells treated with 5 mM NAC for 30 min; BPV: cells treated with 2.0 mM BPV for 60 min; NAC+BPV: cells pretreated with 5 mM NAC for 30 min, following treated with 2.0 mM BPV for 60 min. (**A**, **B**) the fluorescence intensities of mtROS in cells pretreated with NAC, following incubation with BPV; (**C**, **D**) the western blot analysis showed JNK phosphorylation in cells pretreated with NAC, following incubation with BPV; (**E**, **F**) the western blot analysis showed SOD2 transcription in cells pretreated with NAC, following incubation with BPV; (**G**) LDH production in cells treated with 2.0 mM BPV for 60min and/or pretreated with 5 mM NAC for 30min. Values are the mean± SEM of n = 3, *: *P*< 0.05.

N-acetyl-L-cysteine (NAC, a ROS scavenger) was employed to determine the effect of mtROS on JNK-SOD2 signaling. The results showed that NAC significantly reduced mtROS production, the increase of JNK phosphorylation and SOD2 transcription in group NAC (vs. group Con, *P*< 0.05). It also significantly reduced BPV-induced mtROS generation, the increase of JNK phosphorylation and SOD2 transcription (group NAC+BPV vs. group BPV, *P*< 0.05); At the same time, NAC significantly attenuated BPV-induced neurotoxic injury (group NAC+BPV vs. group BPV, *P*< 0.05). (Figure 3)

**Figure 4:**
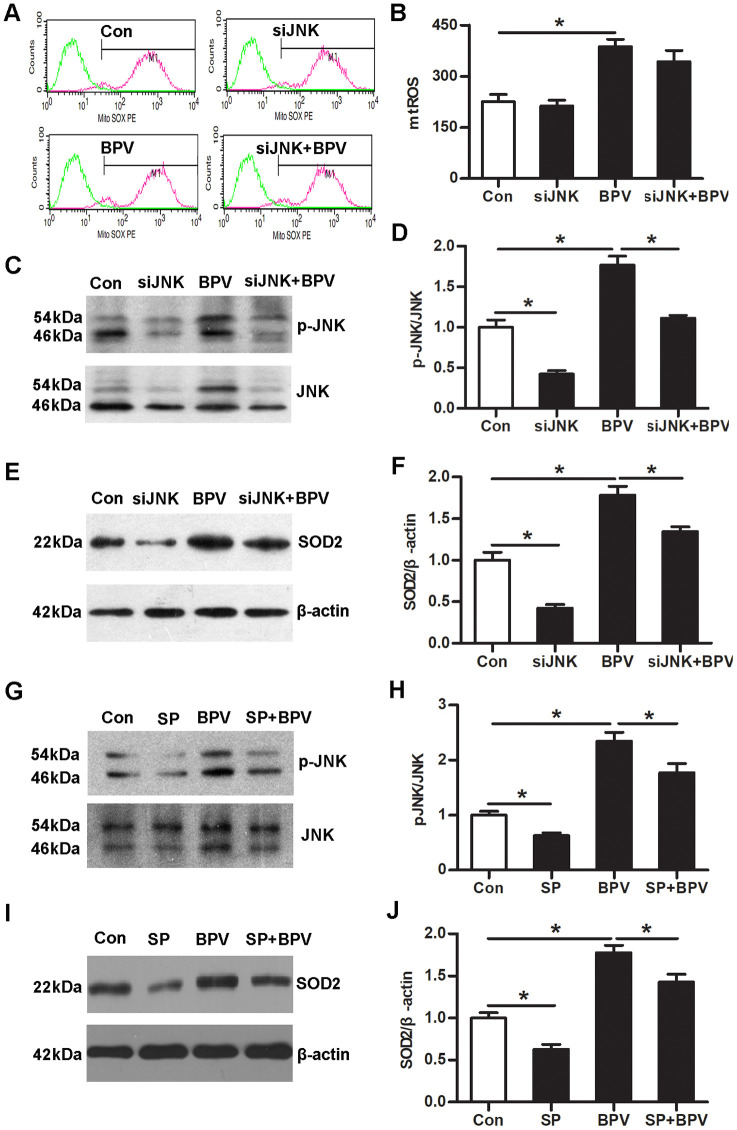
**JNK signaling up-regulated of SOD2 transcription in BPV-induced neuron oxidative stress.** (**A**–**F**) the effect of JNK gene knock-down on the mtROS production, JNK phosphorylation and SOD2 transcription in cells treated with BPV. Con: cells transfected with silencer negative control siRNA; siJNK: cells transfected with JNK siRNA. BPV: cells transfected with silencer negative control siRNA and treated with 2.0 mM BPV for 60 min; siJNK+BPV: cells transfected with JNK siRNA and treated with 2.0 mM BPV for 60 min. (**G**–**J**) The effect of sp600125 on JNK activation and SOD2 transcription in cells treated with BPV; Con: untreated cells; SP: cells cultured with 10 μM sp600125 for 30 min; BPV: cells treated with 2.0 mM BPV for 60 min; SP+BPV: cells precultured with 10 μM sp600125 for 30 min, following treated with 2.0 mM BPV for 60 min. Values are the mean± SEM of n = 3, *: *P*< 0.05.

Next, small interfering RNA (siRNA) and sp600125 (an inhibitor of JNK signaling) were employed to confirm the mechanic of JNK signaling in SOD2 transcription. Down-regulation of JNK expression significantly decreased SOD2 transcription in cells (group siJNK vs. group Con, *P*< 0.05). Simultaneously, the same effect can be achieved at inhibiting activation of JNK signaling (group SP vs. group Con, *P*< 0.05). More importantly, SOD2 transcription was significantly inhibited in group siJNK+BPV or group SP+BPV (vs. group BPV, *P*< 0.05). However, JNK gene knock-down had no effect on BPV-induced mtROS production (group siJNK+BPV vs. group BPV, *P*> 0.05). (Figure 4)

### SOD2 gene knock-down enhanced BPV-induced mtROS over-production, oxidative injury and apoptosis in vitro

**Figure 5:**
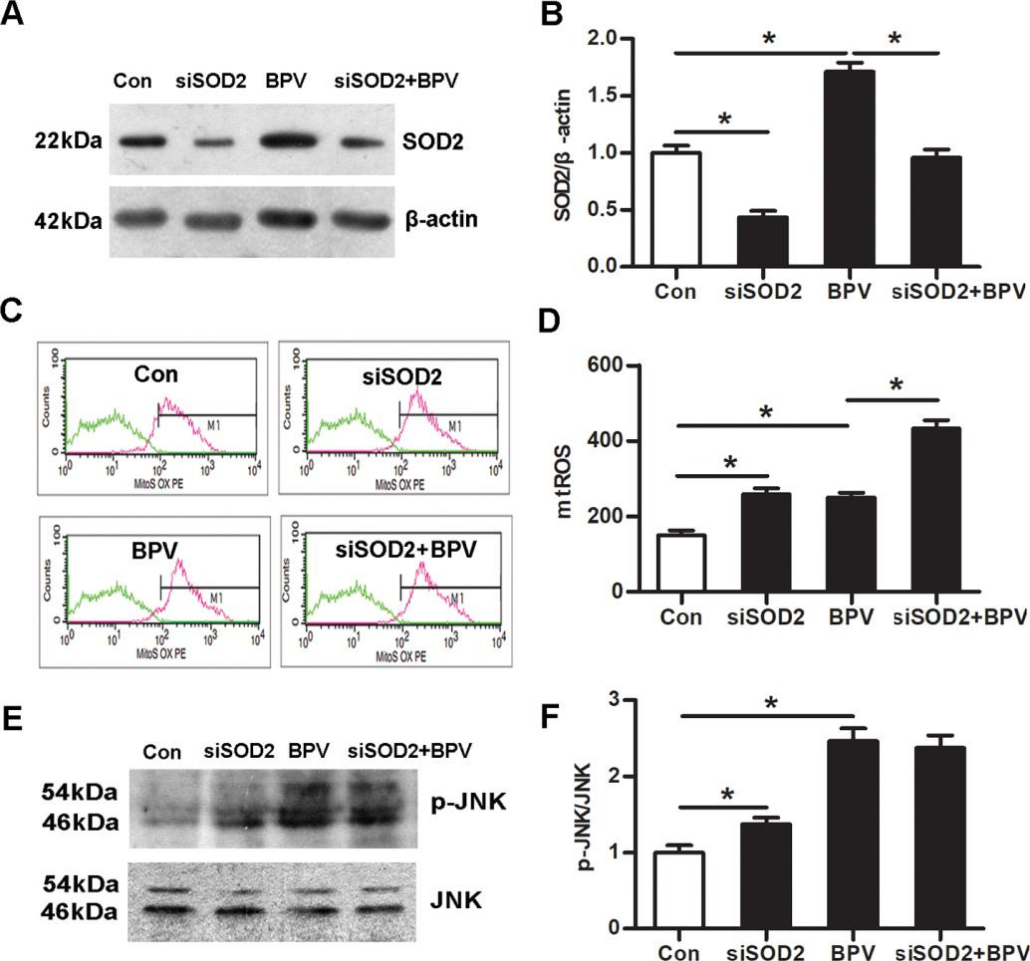
**The effect of SOD2 gene knock-down on mtROS-JNK signaling in BPV-induced neuron oxidative stress.** Con: cells transfected with silencer negative control siRNA; siSOD2: cells transfected with SOD2 siRNA. BPV: cells transfected with silencer negative control siRNA and treated with 2.0 mM BPV for 60 min; siSOD2+ BPV: cells transfected with SOD2 siRNA and treated with 2.0 mM BPV for 60 min. (**A**, **B**) the western blot analysis showed SOD2 in cells transfected with siRNA and treated with BPV; (**C**, **D**) mtROS was monitored by flow cytometry which showed the effect of knockdown SOD2 on mtROS in cells transfected with siRNA and treated with BPV. (**E**, **F**) the western blot analysis showed the activation of JNK signaling in cells transfected with SOD2 siRNA and treated with BPV. Values are the mean± SEM of n = 3, *: *P*< 0.05.

Cells were transfected with SOD2 siRNA or negative control siRNA. SOD2 gene knock-down increased mtROS generation in cells (group siSOD2 vs. group Con, *P*< 0.05). Meanwhile, it also enhanced BPV-induced mtROS over-production (group siSOD2+BPV vs. group BPV, *P*< 0.05). (Figure 5A–5D)

Further, the effect of SOD2 gene knock-down on JNK phosphorylation was determined. The results showed that SOD2 gene knock-down elevated JNK phosphorylation in group siSOD2 (vs. group Con, *P*< 0.05). However, it had no effect on JNK phosphorylation in group siSOD2+BPV (vs. group BPV, *P*> 0.05). (Figure 5E and 5F)

**Figure 6:**
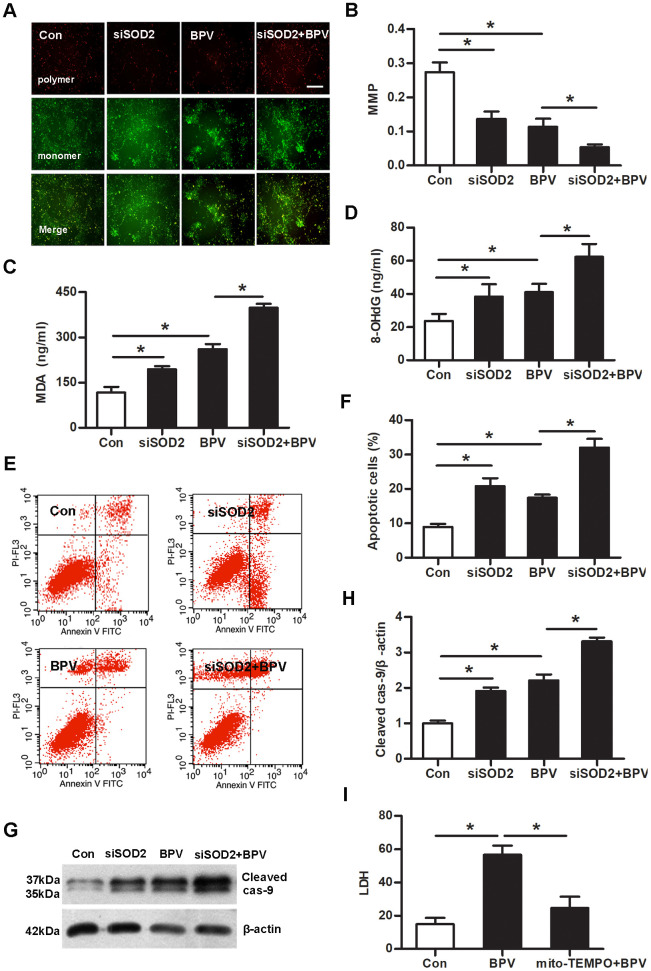
**SOD2 gene knock-down enhanced BPV-induced neurotoxic injury and mito-TEMPO attenuated above injury.** Con: cells transfected with silencer negative control siRNA; siSOD2: cells transfected with SOD2 siRNA. BPV: cells transfected with silencer negative control siRNA and cultured with 2.0 mM BPV for 60 min; siSOD2+BPV: cells transfected with SOD2 siRNA and cultured with 2.0 mM BPV for 60 min. (**A**, **B**) MMP was monitored by determining the relative amount of dual emissions from mitochondrial JC-1 monomers or aggregates using flow cytometry. Mitochondrial depolarization is indicated by a decrease in the polymer/monomer fluorescence. scale bar 200 μm. (**C**, **D**) MDA and 8-OHdG were detected by ELISA; (**E**–**H**) cells apoptosis was detected with flow cytometry and cleaved caspase-9 expression; (**I**) LDH production in cells treated with 2.0 mM BPV for 60 min and/or pretreated with 25 μM mito-TEMPO for 60 min. Values are the mean± SEM of n = 3, *: *P*< 0.05.

Mitochondrial depolarization was measured with JC-1 staining. Mitochondrial membrane potentials (MMP) was declined in group siSOD2 or group BPV (vs. group Con, *P*< 0.05). SOD2 gene knock-down significantly aggravated BPV-induced the decrease of MMP (group siSOD2+BPV vs. group BPV, *P*< 0.05). MDA and 8-OHdG production were increased in group siSOD2 or group BPV (vs. group Con, *P*< 0.05). This effect was further exacerbated in group siSOD2+BPV (vs. group BPV, *P*< 0.05). The cells apoptosis was detected by flow cytometry and cleaved caspase-9 expression. Cells apoptosis was elevated in group siSOD2 or group BPV (vs. group Con, *P*< 0.05). SOD2 gene knock-down significantly enhanced BPV-induced cells apoptosis (group siSOD2+BPV vs. group BPV, *P*< 0.05). As a control antioxidant in mitochondria, mito-TEMPO was used to protect cells against oxidative injury and 25 μM mito-TEMPO performed significant oxidation resistance in SH-SY5Y cells as previously described [23]. So, this concentration of mito-TEMPO was used to preculture cells and it could attenuate BPV-induced neurotoxic injury (group mito-TEMPO+BPV vs. group BPV, *P*< 0.05). (Figure 6)

## DISCUSSION

The purpose of this study is to characterize SOD2 in BPV-induced neuron oxidative stress and find out its mechanistic pathway. There are three main findings in the present study. First, BPV caused neuron oxidative stress with concomitantly stimulating activation of JNK signaling and SOD2 transcription. Second, mtROS-JNK signaling was a co-regulator of SOD2 transcription in BPV-induced neurotoxic injury. Third, SOD2 deficiency enhanced BPV-induced neurotoxicity and mito-TEMPO protected cells against above injury.

Local anesthetics are widely used in regional anesthesia and analgesia, and have certain neurotoxic effects on neuron. Previous studies have confirmed that local anesthetics can trigger intracellular Ca^2+^ homeostasis imbalance, mitochondrial and endoplasmic reticulum oxidative stress. A large amount of ROS production is a crucial factor in local anesthetics-induced toxic injury such as BPV, lidocaine and ropivacaine [9, 24, 25]. However, previous studies focus on cell oxidative injury and whether the results of vivo model are consistent with above changes is not clear. In this study, vivo injury model was developed and the results showed BPV caused spinal cord oxidative stress and apoptotic injury, and subsequent spinal reflex dysfunction. Next, BPV was used to culture SH-SY5Y cells and the results also demonstrated that it stimulated mtROS production in a concentration- and time-dependent manner with parallel cellular toxic injury. SOD2 resides predominantly in the mitochondrial matrix as a key antioxidant enzyme. It was found also in nucleoid complexes with mitochondrial DNA to protect them from oxidative injury and inactivation respectively. Amazingly, SOD2 appears to subject to inactivation in response to oxidative stress, and tyrosine nitration [26]. In contrast, we found that SOD2 transcription was up-regulated in BPV-induced neuron oxidative stress. The mechanism governing the interaction of mtROS production and SOD2 transcription in above oxidative injury model is not clear.

Cells survival or death is closely related to the activation of JNK signaling which modulates stress related pathway in oxidative injury [27]. SOD2 transcription through mtROS-driven JNK signaling has not been explored. In the present study, we investigated JNK phosphorylation and SOD2 transcription. The results showed that the activation of JNK and SOD2 transcription were increased in response to BPV-induced oxidative stress. So, we speculated that BPV could stimulate mtROS production, following activated JNK signaling which up-regulated SOD2 transcription. Antioxidant NAC was used to inhibit mtROS production for confirm its effect on the activation of JNK signaling and SOD2 transcription. The results showed that NAC effectively reduced mtROS production, antagonized the JNK phosphorylation and down-regulated SOD2 transcription. Further, JNK gene knock-down inhibited BPV-induced the up-regulation of SOD2 transcription. At same time, a JNK inhibitor (sp600125) also decreased JNK phosphorylation and down-regulated SOD2 transcription. Above evidence suggested that the activation of JNK signaling may be responsible for mtROS-stimulated SOD2 transcription in BPV-induced neuron oxidative stress.

Cells lose the capacity for being adequate to scavenge ROS contributing to mitochondria oxidative stress and apoptosis pathway [28]. The scavenging ability of SOD2 plays a critical role in maintaining the dynamic balance of ROS production and the integrity of mitochondria. SOD2 deficiency shows a variety of mitochondrial abnormalities such as reduced complex I and II activities [26, 29], enhanced lipid peroxidation and increased oxidative stress [30]. Over-expression of SOD2 decreases lipid peroxidation and mtROS generation, subsequently inhibits oxidative stress and cells death [31]. Mitochondrial depolarization is a critical and relatively early event in mitochondrial oxidative injury process, which eventually leads to mitochondrial permeability transition and subsequent apoptosis [32]. For determining the role of SOD2 in BPV-induced neurotoxic injury, siRNA was used to knock down SOD2 gene. The results showed that SOD2 gene knocke-down enhanced BPV-induced mtROS production, accompanied with MMP declining and apoptotic injury. Further, mito-TEMPO, a mitochondrial antioxidant, could protect cells against BPV-induced neurotoxic injury. Above results suggested that activation of SOD2 could attenuate BPV-induced oxidative stress.

Some limitations should be noted that BPV application in clinical is 0.5%-0.75%. For building spinal cord injury model, 2.5% BPV was used for intrathecal injection in rats as previously described [33], which was greater than clinical doses. In vitro injury model, BPV concentration was 2 mM, which was equal to 0.06% clinical concentration. According to previous report, almost all SH-SY5Y cells cultured with clinical concentration of BPV will be killed [34]. In clinic, the axons in the nerve roots of the cauda equina suffer from the brunt of BPV intrathecal injection which is greater than the cells culture concentration. So, BPV-induced neurotoxic injury can be confirmed in vivo and vitro models.

In summary, our study reveals that mtROS-JNK signaling is a co-regulator of SOD2 transcription in BPV-induced oxidative stress. Enhancing the antioxidant ability of SOD2 might be employed as a promising therapy in the prevention of BPV-induced neuron oxidative injury.

## MATERIALS AND METHODS

### Surgical procedure and group assignment of rats

This study was approved by the Animal Research Center of Southern Medical University (protocol number: SYXK-2016-0167, Guangzhou, China), which follows the Guide for the Care and Use of Laboratory Animals (NRC1996). Animal experiments were conducted in male Sprague-Dawley rats (8-10 weeks old, 250-300 g) from Animal Research Center of Southern Medical University. All rats received intrathecal catheterization as described previously [33]. Rats were allowed at least 2 days to rest for recovery from the operation. Rats with tail movements or motor dysfunction in the hind limbs were not used in next experiments. For building BPV-induced rat spinal cord injury model, we used 2.5% BPV as previously described [33]. Under sevoflurane anesthesia (1.5%) with oxygen and room air via a nose cone, 2.5% BPV hydrochloride (dissolved in 0.9% saline) or 0.9% saline was injected intrathecally at the L_5_- L_6_ intervertebral space using a 50gauge needle. The volume of the injectate was 0.2 μl/g body weight.

For investigating the effect of BPV on oxidative damage in spinal cord, rats were divided into four groups: group con (rats after injection of 0.9% saline with 0.2 μl/g, n= 6), group 6 h (rats at 6 h after injection of 2.5% BPV with 0.2 μl/g, n= 6), group 12 h (rats at 12 h after injection of 2.5% BPV with 0.2 μl/g, n= 6), and group 24 h (rats at 24 h after injection of 2.5% BPV with 0.2 μl/g, n= 6). Following, all rats´ spinal cord tissues (about 10 mm intumescentia lumbalis) were prepared as described [35].

### Spinal reflex function in rats

Spinal cord function was assessed by evaluating hind limb withdrawal reflex responses to mechanical and thermal stimuli with calculating the PWT (g) and TWL (s) in different times (before injection, 6^th^ h, 12^th^ h and 24^th^ h after BPV injection), with the experimenter blinded to initial treatment group as previously described [36].

### Cells culture and transfection

The human neuroblastoma SH-SY5Y cell line was purchased from the Shanghai Institutes for Biological Sciences (Shanghai, China). Cells were cultured in DMEM/F12 medium (Gibco, Grand Island, NY) supplemented with 10% FBS (Gibco, Grand Island, NY) and 1% penicillin/streptomycin at 37°C in 5% CO_2_. The culture medium was replaced every two days. Cells were grown in 100-mm dishes and sub-cultured in 6-well (seeding density 5.0 × 10^5^ cells), 12-well (seeding density 1.0 × 10^5^ cells). Experiments were conducted when cells reached 85% confluence. BPV hydrochloride (purity 99.9%), NAC and mito-TEMPO (Sigma, St. Louis, MO) were dissolved in the media. Sp600125 (Enzo Life Sciences, Farmingdale, NY, USA) was dissolved in dimethyl sulfoxide (DMSO) (Sigma, St. Louis, MO). JNK siRNAs (5´-GAUGGAAACGACCUUCUAUdTdT-3´ and 5´-AUAGAAGGUCGUUUCCAUCdTdT-3´), SOD2 siRNAs (5´-GUUGGCUUGGUUUCAAUAAdTdT-3´ and 5´-UUAUUGAAACCAAGCCAACdTdT-3´) and Silencer Negative Control siRNA (5´-UUCUCCGAACGUGUCACGUTT-3´ and 5´-ACGUGACACGUUCGGAGAATT-3´) were from Sigma (St. Louis, MO). Transfection was performed according to manufacturer instructions.

### Measurement of LDH

LDH (a maker of cell toxic injury) in culture medium was detected with Assay Kit (Beyotime Biotechnology, China) according to the manual instructions as described [37]. The amount of LDH from injury cells was quantified using absorbance captured at 490 nm.

### Measurement of MDA and 8-OHdG

As described in previous study [38], rat spinal cord and cell DNA were extracted with DNeasy Blood and Tissue Kit (Qiagen, Germany) according to manufacturer instructions. MDA and 8-OHdG production were determined using an ELISA kit (R&D Systems, MN, USA) according to kit instructions. The average MDA and 8-OHdG concentration per microgram of protein for each experimental group was calculated.

### Western blot assay

Preparation of lysates, determination of protein concentrations, electrophoresis, and immunoblotting were conducted as previously described [9]. They were immunoblotted with anti-SAPK/JNK (1:500, CST, Danvers, MA), anti-phospho-SAPK/JNK (Thr183/Tyr185) (1:500, CST, Danvers, MA), anti-SOD2 (1:500, CST, Danvers, MA), anti-caspase-3 (1:500, CST, Danvers, MA), anti-caspase-9 (1:500, CST, Danvers, MA) or anti-β-actin (1:1,000, Sigma, St. Louis, MO) diluted in blocking solution containing 5 % nonfat dry milk and 0.1 % Tween-20 in Tris-HCl-buffered saline overnight. The immunocomplexes were visualized using chemiluminescence. Band densities were measured using a densitometer and analyzed with Quantity One analysis software (Bio-Rad, Hercules, CA). Relative protein expression levels were normalized to corresponding β-actin bands.

### Detection of mtROS

Measurement of mtROS was performed using MitoSOX (Invitrogen, Carlsbad, CA). Briefly, after drugs treatment in 6-well plates, cells were incubated with 5 μM MitoSOX at 37 °C for 15 min, then collected and washed three times with phosphate buffer saline (PBS). The concentration of MitoSOX at 5 uM was chosen based on previous study conducted in SH-SY5Y cells [23], and our preliminary experiments which showed that the suitability to catch oxidative stress in our experimental settings. Measurement was performed using flow cytometry (BD FACS Calibur, BD Biosciences, USA) at excitation/emission wavelengths of 510/580 nm.

### MMP assay

Mitochondrial depolarization was measured with JC-1 assay kit (Life Technologies Corporation, USA). After treatment, cells were incubated for 20 min with JC-1 staining solution (5 μg/ml) at 37 °C and rinsed twice with PBS. MMP was monitored by determining the relative amount of dual emissions from mitochondrial JC-1 monomers or aggregates using flow cytometry [39]. In injury cells with low MMP, JC-1 remains in the monomeric form and shows only green fluorescence. Mitochondrial depolarization is indicated by a decrease in the polymer/monomer fluorescence intensity ratio.

### Apoptosis assay by flow cytometry

After treatment in 24-well plates, cells were rinsed with PBS, and resuspended in 500 μl binding buffer. Annexin V-FITC (5 μl) and propidium iodide (5 μl) (KeyGEN, Nanjing, China) were added in cells following suspension. After a 10 min incubation, cell apoptosis was determined by flow cytometry (BD FACS Calibur, BD Biosciences, NJ, USA). Apoptosis are Annexin V-FITC positive and PI-negative (statistics of upper right quadrant and lower right quadrant).

### Statistical analysis

Data are presented as means ± standard error of the means (SEMs). Statistical differences of multiple groups were calculated by multiple comparisons with variance analysis, followed by Turkey's post hoc test. Differences between two groups were calculated by two tailed unpaired or paired Student´s t test. Statistical analysis was performed with SPSS soft-ware 13.0 (SPSS Inc., Chicago, IL). significance was set at *P*< 0.05.
